# S-factor of ${}^{14}N(\alpha \text{,}\gamma ){}^{18}F$ reaction at low-energies

**DOI:** 10.1016/j.rinp.2018.04.018

**Published:** 2018-06

**Authors:** H. Khalili

**Affiliations:** Department of Physics, Faculty of Sciences, Arak University, Arak 38156-8-8349, Iran

**Keywords:** Radiative capture, The astrophysics S-factor, Potential model

## Abstract

The astrophysical S-factor of the ${}^{14}N(\alpha \text{,}\gamma ){}^{18}F$ reaction has been studied at range of bombarding energy 1–1.30 MeV. The ${}^{14}N(\alpha \text{,}\gamma ){}^{18}F$ process is important in low energy astrophysics so that a possible source of energy in massive stars which have spent their hydrogen cycle. Using the Wood-saxon potential model, we have been calculated non resonances the astrophysical S-factors for the E2 transition and our results for $E_{\alpha }=0.0$ MeV is S≈0.5 MeV.b where from experimental is measured to $E_{\alpha }=0.0$ is S≈o.7 MeV.b (Couch et al., 1971) that in comparison with our data good agreement is achieved for the astrophysical S-factor of this process.

## Introduction

The ${}^{14}N(\alpha \text{,}\gamma ){}^{18}F$ reaction is one of the processes of interest in nuclear astrophysics. furthermore, Nitrogen isotopes especially ${}^{14}N$ is used in the Carbon-Nitrogen-Oxygen (CNO) hydrogen-burning cycle. Therefore ${}^{14}N$ density is high in around stars. Because of the relatively great abundance of ${}^{14}N$, alpha capture by ${}^{14}N$ will also be important in the nucleosynthesis which places in hydrogen-depleted helium regions under condition of explosive-burning [2]. The ${}^{14}N$ be used at great temperatures in this reaction and the rate for ${}^{14}N(\alpha \text{,}\gamma ){}^{18}F$ reaction will be most critical in the area $T_{9}⩾0.5$.

The ${}^{14}N(\alpha \text{,}\gamma ){}^{18}F$ reaction is important in *He* burning, taking place before the triple-α reaction. It is an important source of ${}^{22}\mathrm{Ne}$, with another α capture, which is a neutron source for the *s*-process ${}^{22}\mathrm{Ne}{\left(\alpha \text{,}n\right)}^{25}\mathrm{Mg}$. The ${}^{14}N(\alpha \text{,}\gamma ){}^{18}F$ reaction is a substitute source of energy for the mass low stars where degenerated helium-burning [3], [4]. Developmental path of the red giant phase stars, so that by the end of the helium flash. The explosive nature of the helium flash is decaying matter arises. When the temperature reaches $\approx 10^{8}\;\text{K}$, and helium fusion starts with the $3\alpha \to^{12}C$ process, the temperature rapidly increases, greater raising the rate of fusion of helium-as material rotten good conductor of heat, the development of the reaction region.

On the other hand, the study of ${}^{18}F$ reaction play an important role about levels of ${}^{18}F$, which has not previously been data. We can surely predict that a more undiscovered level exists, the similarity of the ground states of ${}^{18}F$ [5]. In addition to it determining cross section for ${}^{14}N(\alpha \text{,}\gamma ){}^{18}F$ reaction in low energy there are a number of excited states in ${}^{18}F$ which could be essential as low energy resonances in this process [6]. Price in the ${}^{14}N(\alpha \text{,}\gamma ){}^{18}F$ reaction at α-particle energies of 1.53 and 1.62 MeV, and have been calculated through the same reaction by a number of other researcher. Although, several of other works has searched at low energy about E=0.64 MeV, no other resonances were found lower than 2 MeV [6].

In resonance relates to a level of spin and parity $J^{\pi }=2$ in the ${}^{18}F$ composite nucleus, which was before detected at an excitation energy $E_{x}=5786\pm 2.4$ keV via the ${}^{14}N(\alpha \text{,}\gamma ){}^{18}F$ reaction [7], [8]. In some experiments,the lifetime of this state was found to be τ=15±10 fs from a amount based on the Doppler-shift reduction method [7]. This quantity led to the slightly ratio $\Gamma_{\gamma }/\Gamma_{\alpha }∼1$, which was used for approximating the (p,α) resonance power [9], [10].

The reaction rate of the ${}^{14}N(\alpha \text{,}\gamma ){}^{18}F$ reaction is dominated by the contribution of a $J^{\pi }={\left(1\right)}^{-1}$ resonance at an a energy of 572 keV(in the laboratory system) for temperatures of astrophysical interest, T=0.1-5 GK. Higher energies (51136 keV, 1398 keV, 1527 keV, 1529 keV, and 1618 keV) for higher temperature become more important. For lower temperature (about ∼0.1 GK) contributions are from the low-energy sequence of the 572 keV resonance and the $J^{\pi }=4^{+}\text{,}T=1$ resonance at 305 keV [14], [10], [11].

The electromagnetic transition (E2(M2)) strength of 25 ± 7(600 ± 130) W.u. for the 5298$\left(4^{+}\right)$ ⟶ $2523(2^{+})$ keV transition the P(1) substrate should be populated in the ${}^{14}N(\alpha \text{,}\gamma ){}^{18}F$ reaction (J), in good agreement with the decoction of P(1)>90% and P(0)<10% . This $J^{\pi }$ obligation demands that the 5298 keV resonance state is molded by g-wave capture. The α-width of $\Gamma_{\alpha }=10\pm 4$ MeV matches to a decreased with $\theta^{2}∼0.54$ for an collaboration range of 4.8 fm [7].

In 1971 by Couch et al. total cross section calculated for ${}^{14}N(\alpha .\gamma ){}^{18}F$ reaction for $0.5⩽E_{\alpha }⩽1.2$ MeV and at 0.56 MeV related to energy of α, a new resonance has been found corresponding to the 4.849 MeV state in ${}^{18}F$. Theoretical studies of direct radiative capture for cross section of $E_{\alpha }=0.0$, for the non-resonant S-factor a value S≈0.7 MeV.b has been estimated [1]. Bertulani et al., more recently, present computer RADCAP code at calculations of observables for nuclear reaction at low energy [13]. We have been used this code and calculated astrophysical S-factor of ${}^{12}C{\left(\alpha \text{,}\gamma \right)}^{16}O$ astrophysics S-factor radiative capture [18]. Also, we have been used this code for calculation astrophysical S-factor ${}^{14}N(\alpha \text{,}\gamma ){}^{18}F$ reaction.

This paper is organized as follows: In Section ‘Brief review of theoretical framework’ we study the wood-saxon potential and the formalism cross section of the alpha-nitrogen(14) radiative capture. We discuss the theoretical errors, tabulation of the calculated cross section in comparison with the other theoretical approaches and the available experimental data in Section ‘Results and discussion’. Finally, summary and conclusions follow Section ‘Summary and conclusions’.

## Brief review of theoretical framework

We this work have used the computer RADCAP code for radiative capture of ${}^{14}N(\alpha \text{,}\gamma ){}^{18}F$ reaction and calculate various quantities this reaction. The spacial part wave functions of ${}^{18}F$ are explained by $\psi_{\mathrm{JM}}\left(r\right)$:

(1) $$\psi_{\mathrm{JM}}\left(r\right)=\frac{1}{r}U_{\mathrm{lj}}^{J}Y_{\mathrm{JM}}^{l}\text{,}$$

where *r* is the relative coordinate of a(α) and $b({}^{14}N)$, $U_{\mathrm{lj}}^{J}(r)$ is the radial wave function and $Y_{\mathrm{JM}}^{l}$ is the angle-spin wave function by Clebsch-Gordan coefficients($<{\mathrm{jmI}}_{a}M_{a}|\mathrm{JM}>$)

(2) $$Y_{\mathrm{JM}}^{l}=\underset{m.M_{a}}{\sum }<{\mathrm{jmI}}_{a}M_{a}|\mathrm{JM}>|I_{a}M_{a}>|\mathrm{jm}>$$

where |jm>=∑ml,Mbylml(r^)XMb and $X_{M_{b}}$ is the spin wavefunction of particle ${}^{14}N$. The wave functions are evaluated using the $V_{o}(r)\text{,}V_{S}(r)$ and $V_{C}(r)$ that are central, spin-orbitand the Coulomb potentials respectively. The potentials $V_{o}(r)$ and $V_{S}(r)$ are given by

(3) $$\begin{array}{cc} & V_{o}(r)=v_{o}\;R_{o}(r)\text{,}\\ & V_{S}(r)=-\;\frac{\hbar^{2}v_{\mathrm{So}}}{m_{\pi }^{2}c^{2}r}\;\frac{d}{\mathrm{dr}}R_{\mathrm{So}}(r)\\ & R_{i}(r)=\frac{1}{\left[1+\exp\left(\frac{r-R_{i}}{a_{i}}\right)\right]} \end{array}$$

where $v_{o}\text{,}v_{\mathrm{So}}\text{,}R_{o}\text{,}a_{o}\text{,}R_{\mathrm{So}}$, and $a_{\mathrm{So}}$ are moderated so that the ground state energy *Q* or the energy of an excited state, is reproduced. We can studied the bound state wave functions with solving the radial Schrödinger equation

(4) $$-\frac{\hbar^{2}}{2\mu }\left[\frac{d^{2}}{{\mathrm{dr}}^{2}}-\frac{l\left(l+1\right)}{r^{2}}\right]U_{\mathrm{lj}}^{J}\left(r\right)+\left[V_{o}\left(r\right)+V_{C}+\left(r\right)+\langle s.l\rangle \;v_{\mathrm{So}}\left(r\right)\right]U_{\mathrm{lj}}^{J}\left(r\right)=E_{i}U_{\mathrm{lj}}^{J}\left(r\right)$$

where spin–orbit interaction is explained by $\langle s.l\rangle =\frac{1}{2}\left[j(j+1)-s(s+1)-l(l+1)\right]$ and $E_{i}$ are discrete energies relevant to bound state and coulomb potential. The magnetic and electric transitions are studied by [12].

(5) O^e=eeffλrλyλμr^,eeffλ=Zbe-mamcλ+Zaembmcλ,O^m=34πμNeeffMlμ+∑i=a,bgisiμ,eeffM=ma2Zamc2+mb2Zbmc2.

$e_{\mathrm{eff}}^{\lambda }$ and $e_{\mathrm{eff}}^{M}$ are effective electric and magnetic charges, respectively. The orbital and spin angular momentum,$l_{\mu }$ and $s_{\mu }$, are the spherical components of order μ(μ=-1,0,1) and $g_{i}$ are the gyromagnetic factors of input particles(α) and $\mu_{N}$ is nuclear magneton.The matrix element of quadropole moment for this transition is given by Wigner-Eckart thoeorem [12]

(6) JMO^eJ0M0=J0M0λμ|JMJO^eJ02J+1,JO^eJo=-1j+Ia+Jo+λ2J+12Jo+11/2jJIaJ0joλljO^elojoJ,

For lo+l+λ=even,ljO^elojoJ,is given by

(7) ljO^elojoJ=eλ4π-1lo+l+jo-jλ^jo^ȷ^<jo12λ0|j12>∫o∞drrλUljJrlojoJor.

For $l_{o}+l+\lambda =\mathrm{even}$, the reduced matrix elements is not null. At very low energies,the magnetic transitions(M1) will be much smaller than the electric transitions in the cross section of photo radiative capture but the magnetic transitions M1 plays an important role in the case of sharp resonances [16]. The reduced matrix elements for magnetic dipole of transition, in the case of $l=l_{o}$ (for $l\;\neq \;l_{o}$ the magnetic moment matrix element of M1 is zero), is given by [17]

(8) ljO^mlojoJ=-1j+Ia+Jo+134πJ^J^ojJIaJojo1μN×1l^oeM2j∼ol^oloδjo,lo+1/2+lo+1δjo,lo-1/2+-1lo+1/2-jj^o2δjo,lo±1/2δj,lo∓1/2+gN1l^o2-1lo+1/2-joj^oδj,jo--1lo+1/2-jj^o2δjo,lo±1/2δj,lo∓1/2+ga-1Ia+jo+J+1J^oJ^I^aI∼aIaJjoJoIa1∫o∞drUljJrUlojoJor,

$g_{N}$ = 5.586(−3.826) and the $\mu =g_{a}\mu_{N}$ are geomagnetic factor for the proton(neutron) and the magnetic moment of the core nucleus respectively. In this work, the alpha particle has zero spin, which should be consider in the above relations. To study the structure of the initial state and the final state for after interaction, needed to reduced transition probability $\mathrm{dB}((Q_{E}\text{,}Q_{B})\lambda )/\mathrm{dE}$ of the nucleus, *i* →j+k. Electromagnetic transition $(Q_{E}\text{,}Q_{B})\lambda$ to a final state with momentum ℏk is given by [13]

(9) $$\frac{\mathrm{dB}}{\mathrm{dE}}((Q_{E}\text{,}Q_{B})\lambda \text{,}J_{i}s\to {\mathrm{kJ}}_{f}s)=\frac{2J_{f}+1}{2J_{i}+1}\underset{j_{f}l_{f}}{\sum }{\left|\underset{j_{i}l_{i}j_{c}}{\sum }\langle {\mathrm{kJ}}_{f}j_{f}l_{f}{\mathrm{sj}}_{c}||T((Q_{E}\text{,}Q_{B})\lambda )||J_{i}j_{i}l_{i}{\mathrm{sj}}_{c}\rangle \right|}^{2}\frac{\mu k}{{\left(2\pi \right)}^{3}\hbar^{2}}$$

The electric multipole ($Q_{E}$) is given by

(10) T(QEλμ)=eeff(λ)rλYλμ(r^)e

where $e_{\mathrm{eff}}^{\left(\lambda \right)}$ is the effective charge number. Final and initial wave function are given by [13]

(11) ψi(r→)=〈r→|Jijilisjc〉=1r∑mimc〈jimijcmc|JiMi〉fJijilijc(r)Yjimilis(r^)ϕjcmcψf(r→)=〈r→|k→Jfjflfsjc〉=4πkr∑mfmc〈jfmfjcmc|JfMf〉gJfjflfjc(r)ilfYlfmf∗(k^)Yjfmflfs(r^)ϕjcmc,

The reduced matrix element in Eq. (9)is given by [13]

(12) $$\langle {\mathrm{kJ}}_{f}j_{f}l_{f}{\mathrm{sj}}_{c}||T(Q_{E}\lambda )||J_{i}j_{i}l_{i}{\mathrm{sj}}_{c}\rangle =\frac{4\pi Z_{\mathrm{eff}}^{\left(\lambda \right)}e}{k}D_{J_{i}j_{i}l_{i}}^{J_{f}j_{f}l_{f}}(\lambda {\mathrm{sj}}_{c})\;{\left(-i\right)}^{l_{f}}I_{J_{i}j_{i}l_{i}}^{J_{f}j_{f}l_{f}}(\lambda j_{c})$$

where the coupling coefficient $D_{J_{i}j_{i}l_{i}}^{J_{f}j_{f}l_{f}}(\lambda {\mathrm{sj}}_{c})$ and the $I_{J_{i}j_{i}l_{i}}^{J_{f}j_{f}l_{f}}(\lambda j_{c})$ are given by

(13) $$\begin{array}{cc} & D_{J_{i}j_{i}l_{i}}^{J_{f}j_{f}l_{f}}(\lambda {\mathrm{sj}}_{c})={\left(-1\right)}^{s+j_{i}+l_{f}+\lambda }{\left(-1\right)}^{j_{c}+J_{i}+j_{f}+\lambda }(l_{i}\;0\;\lambda \;0|l_{f}\;0)\sqrt{2j_{i}+1}\sqrt{2l_{i}+1}\sqrt{2J_{i}+1}\times \sqrt{2j_{f}+1}\sqrt{\frac{2\lambda +1}{4\pi }}\left\{\begin{array}{ccc} l_{i} & s & j_{i}\\ j_{f} & \lambda & l_{f} \end{array}\right\}\left\{\begin{array}{ccc} j_{i} & j_{c} & J_{i}\\ J_{f} & \lambda & j_{f} \end{array}\right\}\text{,}\\ & I_{J_{i}j_{i}l_{i}}^{J_{f}j_{f}l_{f}}(\lambda j_{c})=\int_{0}^{\infty }\mathrm{dr}\;g_{J_{f}j_{f}l_{f}}^{j_{c}*}(r)r^{\lambda }f_{J_{i}j_{i}l_{i}}^{j_{c}}(r) \end{array}$$

The Cross-section of Scattering for particles without spin and nonsame is given by:

(14) $$\sigma_{(E\text{,}B)l}^{\mathrm{cap}}(E)=\frac{\pi \hbar^{2}}{2\mu \varepsilon }(2l+1)T_{(E\text{,}B)l}$$

where $T_{(E\text{,}B)l}$ in the above equation is the transition probability. The total cross section for a transition is:

(15) $$\sigma_{\mathrm{cap}}(E)=\underset{i=l}{\sum }(\sigma_{\mathrm{Ei}}^{\mathrm{cap}}(E)+\sigma_{\mathrm{Mi}}^{\mathrm{cap}}(E))$$

The total cross section for a electromagnetic transition is defined as:

(16) $$\sigma_{\mathrm{cap}}(E)=S(E)\frac{1}{E}e^{-2\pi \eta }$$

S(E) and η in this equation are astrophysical factor and Parameters Samrfyld. Astrophysical *S*-factor is a well-define function and it is easier to analyze, therefore in this study we have used it.

## Results and discussion

**Fig. 1:**
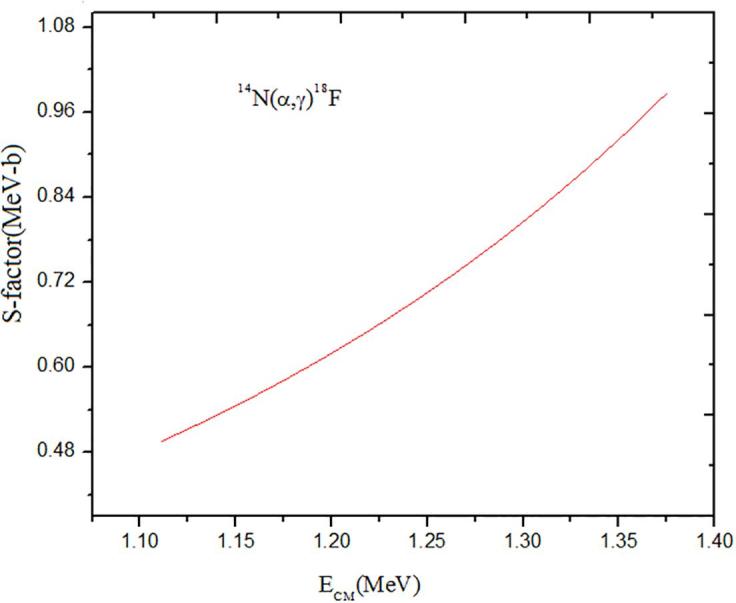
Total astrophysical S-factor of the ${}^{14}N(\alpha \text{,}\gamma ){}^{18}F$ reaction at the range of energies about 1–1.5 MeV.

The astrophysical variation parameters goal in the ${}^{14}N(\alpha \text{,}\gamma ){}^{18}F$ reaction is concerned with its *S*-factor and cross section at low energies.These *S*-factor and cross section can have contributions from nonresonant direct capture at low-energy. The energy dependence of the nonresonant S-factor has been determined by the Coulomb barrier where S-factor independent of energy

(17) $$S=\sigma .E_{\mathrm{CM}}.\mathrm{Exp}\left[\frac{-13.28Z_{1}Z_{2}\mu^{\frac{1}{2}}}{E_{\mathrm{CM}}^{\frac{1}{2}}(\text{KeV})}\right]\text{.}$$

where $E_{\mathrm{CM}}$ is in MeV in the center of mass system and μ is the reduced mass in atomic mass units. The astrophysical S-factor can be used to conclude cross sections to lower energies in the normal way.The previous results of S-factor for the ${}^{14}N(\alpha \text{,}\gamma ){}^{18}F$ reaction was $8.73\times 10^{9}$ keV b [15]. In this paper at α energies of 1.1 and 10390 MeV no signal was found for ${}^{18}F$. We study the radiative capture ${}^{14}N(\alpha \text{,}\gamma ){}^{18}F$ reaction by using RADCAP computer code. In this code, the Schrödinger equation is solved by Wood-Saxon potential model for transition E_2_. The set Wood-Saxon potential parameters is used for solution the Schrödinger equation are given in Table 1. We calculated continuum bound state for this process at range energy in center of mass 1-1.3 MeV and the results nonresonant astrophysical *S*-factor for radiative capture ${}^{14}N(\alpha \text{,}\gamma ){}^{18}F$ process is present in Fig. 1. As be seen at the energy range 1-1.3(MeV),S-factor rise with increase energy. Our results for this reaction have been shown in Table 2. The astrophysics factor based on theoretical results in zero energy ($E_{\alpha }=0.0$) is S≈o.7 MeV.b [1] and our results for $E_{\alpha }=0.0$ MeV is S≈0.5 MeV.b which this model potential is in good agreement with other methods.

**Table 1:** The Wood-Saxon potential parameters are used in our calculations.

$V_{0}(\text{MeV})$	$R_{0}(\mathrm{fm})$	$A_{A}(\mathrm{fm})$	$V_{S0}(\text{MeV})$	$R_{S0}(\mathrm{fm})$	$A_{\mathrm{AS}}(\mathrm{fm})$	$R_{C}(\mathrm{fm})$
−66.6397	2.63391	0.644174	36.8436	2.59506	0.644174	2.63391

**Table 2:** The results of the S-factor ${}^{14}N(\alpha \text{,}\gamma ){}^{18}F$ reaction with the Wood-saxon potential model at low energies.

$E_{\mathrm{cm}}$ (MeV)	S-factor (MeV-b)
1.111	0.4956
1.133	0.5233
1.155	0.5529
1.177	0.5845
1.243	0.6927
1.331	0.8752

On the basis of the experimental of nonresonant yield from the ${}^{14}N(\alpha \text{,}\gamma ){}^{18}F$ reaction in this energy region, the nonresonant *S*- factor for this reaction, $S\leq 1.5\times 10^{6}$ keV-b [6].

## Summary and conclusions

The ${}^{14}N(\alpha \text{,}\gamma ){}^{18}F$ reaction is one of the processes of important in the nucleosynthesis which places in hydrogen depleted helium regions under condition of explosive burning astrophysics. In the past few years, good works have been done with RADCAP code, for example, we have been able to obtain good results for ${}^{12}C{\left(\alpha \text{,}\gamma \right)}^{16}O$ reaction and Sadeghi et al. have been calculated reduced transition probabilities for 4He radiative capture reactions at astrophysical energies that are consistent with experimental data [18], [19].The ${}^{14}N(\alpha \text{,}\gamma ){}^{18}F$ reaction was is investigation with RADCAP code computer. We determined nonresonant S-factor at low energy 1.00-1.40 MeV. Results show that it is less than 1.4 MeV-b and from this theoretical is measured to $E_{\alpha }=0.0$ is nonresonances our results for $E_{\alpha }=0.0$ MeV is S≈0.5 MeV-b. where from experimental is measured to $E_{\alpha }=0.0$ is S≈o.7 MeV.b [1] that in comparison with our data good agreement is achieved for the astrophysical S-factor of this reaction.
